# A Fully Automated Robotic System for Microinjection of Zebrafish Embryos

**DOI:** 10.1371/journal.pone.0000862

**Published:** 2007-09-12

**Authors:** Wenhui Wang, Xinyu Liu, Danielle Gelinas, Brian Ciruna, Yu Sun

**Affiliations:** 1 Advanced Micro and Nanosystems Laboratory, University of Toronto, Toronto, Canada; 2 Program in Developmental and Stem Cell Biology, The Hospital for Sick Children, Toronto, Canada; University of Waterloo, Canada

## Abstract

As an important embodiment of biomanipulation, injection of foreign materials (e.g., DNA, RNAi, sperm, protein, and drug compounds) into individual cells has significant implications in genetics, transgenics, assisted reproduction, and drug discovery. This paper presents a microrobotic system for fully automated zebrafish embryo injection, which overcomes the problems inherent in manual operation, such as human fatigue and large variations in success rates due to poor reproducibility. Based on computer vision and motion control, the microrobotic system performs injection at a speed of 15 zebrafish embryos (chorion unremoved) per minute, with a survival rate of 98% (n = 350 embryos), a success rate of 99% (n = 350 embryos), and a phenotypic rate of 98.5% (n = 210 embryos). The sample immobilization technique and microrobotic control method are applicable to other biological injection applications such as the injection of mouse oocytes/embryos and *Drosophila* embryos to enable high-throughput biological and pharmaceutical research.

## Introduction

Molecule screening at the single cell level, which is critical in molecular biology and drug discovery, requires that target molecules be introduced into single cells to permit cellular-function-targeted molecules to directly regulate cell development and their functions to be quantified. Several technologies exist for introducing foreign materials into a cell, such as electroporation [1], viral vectors [2], gene gun [3], ultrasonics [4], and MEMS-based injection [5]–[6]. Compared to these techniques, microinjection with a single glass micropipette remains the most effective in terms of cell damage, cell viability, cell waste, effectiveness of delivering macromolecules, specificity, and freedom from concerns about phenotype alteration. However, in order to enable fast, precise, and robust screening for molecular targets, the state-of-the-art manual injection must be replaced with fully automated operation.

For testing cellular responses to molecular targets and to obtain statistically significant data, the injection of thousands of cells needs to be conducted within a short time window (e.g., within 1.5 hr after fertilization, before the 16-cell stage for zebrafish embryo injection). Manual injection is not only slow; the laborious task of manual injection easily causes fatigue in injection technicians and hinders performance consistency and success rates. Efforts in automating cell injection have been continuous, resulting in a visually servoed system [7], a semi-automated system [8], and many tele-operated systems [9]–[13], to name just a few. These systems are limited in throughput and reproducibility as operator input (e.g., locating features and destinations) or operator involvement (e.g., switching from one cell to another or injector alignment) is still required.

**Figure 1:**
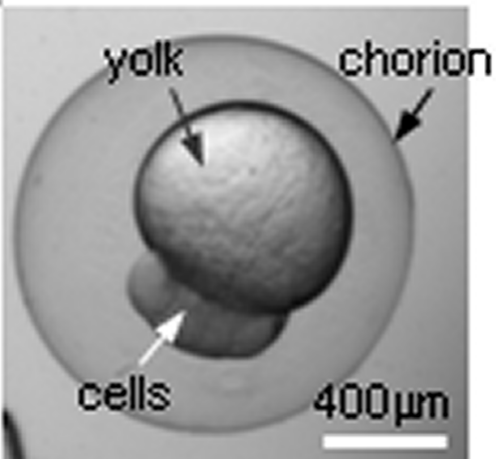
The structure of a zebrafish embryo. Although the embryo is relatively large, it is highly deformable and care must be taken in injection to avoid cell damage.

Among many biological models, the zebrafish has emerged as an important model organism for developmental genetic studies as well as for drug discovery [14]–[15]. Zebrafish embryonic development is remarkably similar to that of humans; however, zebrafish embryos are laid and fertilized externally, they develop rapidly, and the embryos are transparent (Figure 1), making it convenient to observe the movement and fate of individual cells during embryonic development [16]. Molecular and genetic analyses of zebrafish embryogenesis depend on the injection of foreign materials into early zebrafish embryos [17]. DNA injection is used to generate transgenic zebrafish lines, mRNA injection is used to overexpress gene-products in zebrafish embryos, and reverse genetic or loss-of-gene-function studies require the injection of antisense morpholino-modified oligonucleotides (morpholinos or MOs) to specifically inhibit RNA splicing and/or translation *in vivo* [18].

Despite their relatively large size (∼600 µm or ∼1.2 mm including chorion), zebrafish embryos have a delicate structure and can be easily damaged, making automated, high-throughput injection difficult. Specific challenges include: (i) to quickly (i.e., in seconds) immobilize a large number of zebrafish embryos; (ii) to automatically, robustly identify cell structures for vision-based position control and account for size differences across embryos; and (iii) to coordinately control two microrobots to maximize operation speed. Addressing these challenges, the objective of this research was thus to develop an effective massive sample preparation method and create a system that is capable of injecting a large number of embryos in the short time window. In this paper, a microrobotic system for zebrafish embryo injection is presented, featuring full automation, high-speed sample immobilization, and high survival, success, and phenotypic rates.

## Materials and Methods

### Materials

The zebrafish embryos used in the injection experiments were collected in The Hospital for Sick Children (Toronto, Canada) with standard embryo preparation procedures [19]. Animal protocols were approved by the Hospital for Sick Children's Lab Animal Services' Animal Care Committee (protocol #5911). The outbred zebrafish embryos, which were not de-chorionized, were cultured in embryo media that contained 10l reverse osmosis water, 3 g instant ocean salt mix, and 10 ml methylene blue solution (stock = 1 gm/l).

For the ease of visually inspecting the injection effectiveness, fluorescent dyes (Rhodamine B, 100 µM) were injected into 350 embryos. To quantify the efficacy of the system for re-capitulating mutant embryonic phenotypes, fluorescein-tagged morpholinos that target the gene *no tail* (ntl-MO, 5′-GACTTGAGGCAGGCATATTTCCGAT-3′, 300 nM, Gene Tools) were injected into additional 210 embryos. The *no tail* gene product is required for tail formation in zebrafish [20]. Successful injection of ntl-MO should inhibit translation of the *ntl* gene product, resulting in the tail-less phenotype.

Glass capillaries (1.2 mm in outer diameter, TW120F-4, WPI) were heated and pulled using a pipette puller (P-97, Sutter). The tip diameter was 10 µm. The pipette was filled with injection material and connected to a micropipette holder (MPH412, WPI).

### System design

#### System architecture

**Figure 2:**
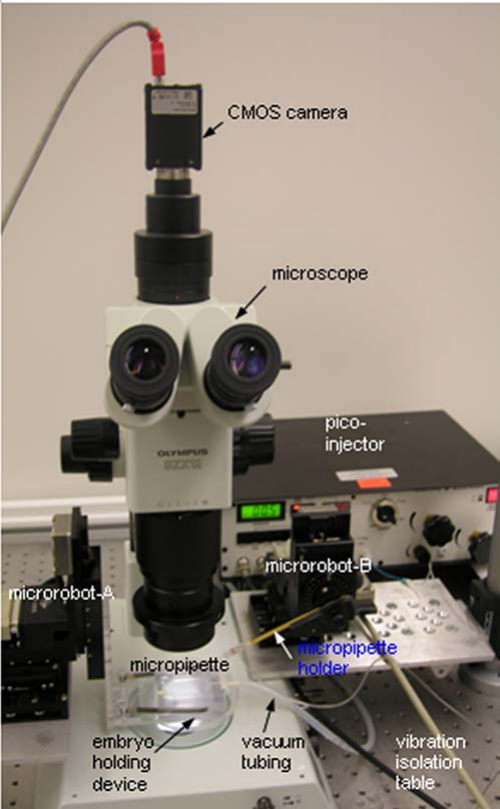
Automatic cell injection system. Microrobot-A and microrobot-B, which are three-degrees-of-freedom motorized micromanipulators with a travel of 25 mm and a 0.04 µm positioning resolution along each axis, control the position of embryos and micropipette, respectively. The system obtains visual feedback through the camera and microscope. The computer-controlled pico-injector provides positive pressure for material deposition.

The system, shown in Figure 2, employs two three-degrees-of-freedom microrobots (MP-285, Sutter) with a travel of 25 mm and a 0.04 µm positioning resolution along each axis. Two motion control cards (NI PCI-6259) are mounted on a host computer (3.0 GHz CPU, 1GB memory) where control algorithms and image processing algorithms operate. Visual feedback is obtained through a CMOS camera (A601f, Basler) mounted on an optical microscope (SZX12, Olympus). An in-house developed embryo holding device is attached to microrobot-A that is used as a precision *XY* stage. A Venturi vacuum pump (UN816, KNF) provides negative pressure to the embryo holding device for immobilizing embryos into regular patterns. The pulled glass capillary is connected to microrobot-B via the micropipette holder. A computer-controlled pico-injector (PLI-100, Harvard Apparatus) provides positive pressure for material deposition. To minimize vibration, all units except the host computer and pressure units are placed on a vibration isolation table.

**Figure 3:**
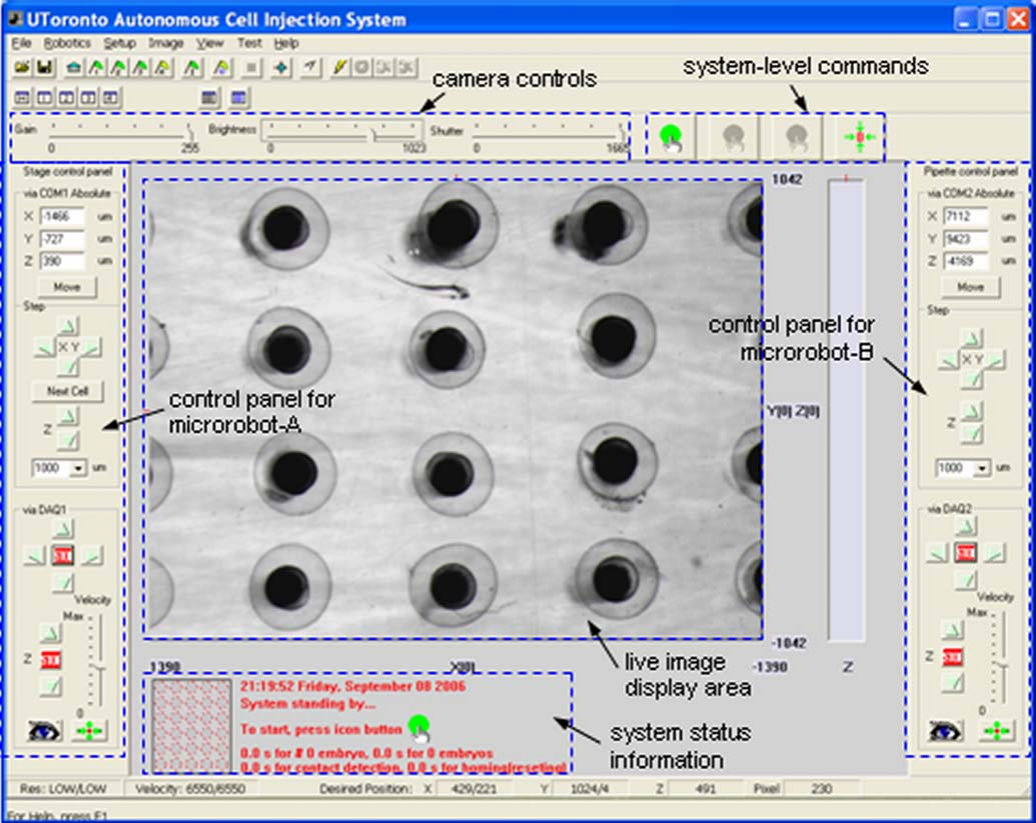
Control program interface with an array of embryos immobilized on the embryo holding device. The embryo image was taken under 0.7×. For fully automated injection, the system-level command buttons enable the user to start, pause/resume, terminate, and reset the system.

Figure 3 shows a screen capture of the control program interface. For fully automated injection, the system-level command buttons permit the user to start, pause/resume, terminate, and reset the system. The live image display area and the system status information window allow for visually monitoring the operation status. The two control panels provide the user with the option for tele-operated injection (i.e., using mouse clicks), alternative to fully automated operation. Users can also readily specify parameters through the control program interface, such as the number of embryos within a batch and camera control parameters.

#### Cell immobilization

**Figure 4:**
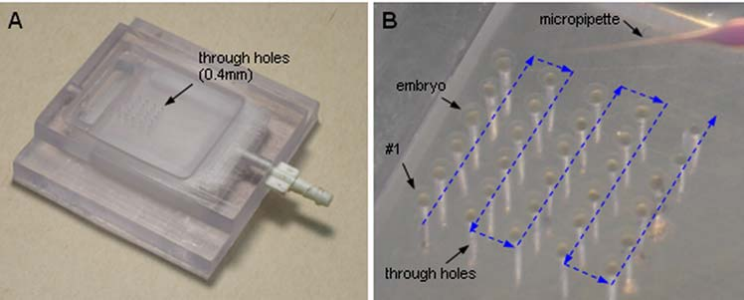
Vacuum-based embryo holding device. Embryos are immobilized on individual through holes via a negative pressure. Extra embryos are flushed off the device. (A) Picture of a device (5×5 holes). (B) An array of immobilized embryos with continuous injection path labeled.

Figure 4 shows the vacuum-based embryo holding device and an array of immobilized zebrafish embryos. Evenly spaced through-holes (diameter ∼400 µm) are connected to a vacuum source via a backside channel. Upon dispersing many embryos onto the device, a sucking pressure enables each through-hole to trap a single embryo. The extra non-trapped embryos are flushed away from the device. In practice, a negative pressure of 2-7 InHg proved effective in immobilizing zebrafish embryos without damaging the embryos. Upon cell immobilization, the system conducts injection continuously along the shortest path (arrow labeled).

#### Volume control

Volume calibration is important for precisely depositing a specified amount of materials into individual cells such that dose effect can be investigated. For the purpose of volume calibration, the automated system pushes a droplet of the material out of the micropipette that forms a sphere at the micropipette tip. Injection volume is then determined by detecting the diameter of the sphere via image processing (Hough transform). According to the calibrated relationship between the injected volume versus pressure pulse level and length, 3 nl materials were deposited into each embryo in the experiments by controlling the pressure ‘on’ time.

### Control flow of automated cell injection

**Figure 5:**
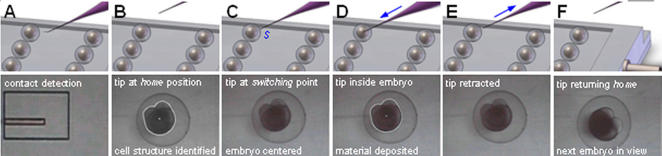
Illustration of the automated injection flow. Except for the task of bringing next embryo into the field of view (from E to F), control of both microrobots is based on “looking-then-moving”. Top row: 3-D view. Bottom row: microscopic (image) 2-D view. (A) The vertical height of the micropipette tip is determined with a computer vision approach. This step is required only once at the beginning of one batch. (B) Micropipette at the *home* position. The white curve outlines the recognized cytoplasm contour. The white dot represents the cytoplasm center. (C) Embryo is brought to the center of the field of view. Micropipette is positioned at the *switching* point. (D) Micropipette tip penetrates the embryo and deposits materials at a pre-set destination in a specified volume. (E) Micropipette retracts out of the embryo. (F) Micropipette returns to the *home* position, and the next embryo is brought into the field of view. From (B) to (C), and from (E) to (F), the two microrobots move in parallel to increase injection throughput.

A batch of zebrafish embryos, immobilized into a regular pattern on the embryo holding device, are placed on microrobot-A under the microscope. Automated injection starts with vision-based contact detection [21] to determine the vertical positions of the micropipette tip and the surface of the embryo holding device (Figure 5) with an accuracy down to 0.2 µm. An embryo is recognized and brought to the center of the field of view; simultaneously, the micropipette tip is moved by microrobot-B to a *switching* point, *S* that serves as an indicator of the boundary between inside and outside of an embryo and is determined through the recognition of embryo structures. The micropipette tip penetrates the chorion and deposits materials at the desired location within the embryo. In the experiments presented in this paper, the deposition destination was chosen to be the cytoplasm center, where *cytoplasm* is defined as the combination of the yolk and the cell portion of a zebrafish embryo. Upon retreating out of the embryo, the micropipette tip is moved to a *home* position that is 1.4 mm above contact point, to prevent it from crashing into the next embryo. In the meanwhile, the next embryo is brought into the field of view, the structures are recognized, and the injection process is repeated until all embryos in the batch are injected.

Throughout the process, microrobot-A does not produce vertical motion while microrobot-B is servoed along three axes, as shown in Figure 5. For positioning each embryo and controlling the motion of the injection micropipette, PID (proportional-integral-derivative) control is employed for controlling both microrobots that are operated in parallel whenever possible (i.e., in Figure 5, from (B) to (C), and from (E) to (F)). Parallel operation of the two microrobots is maximized to increase injection throughput. Transformations among the multiple coordinate frames are achieved during the operation of the system without requiring an off-line process.

### Image processing: Recognizing embryo structures

**Figure 6:**
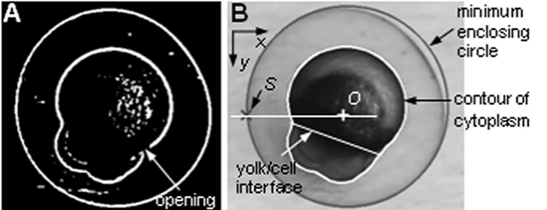
Recognition of zebrafish embryo structures. (A) After pre-processing. (B) Recognized chorion, cytoplasm center, switching point, and yolk/cell interface.

The purpose of recognizing detailed embryo structures is for determining deposition destinations to guarantee a high reproducibility. In this paper, the cytoplasm center (Figure 6B) was chosen as the deposition destination. However, the recognition algorithm allows for choosing a different destination, for example, closer to the yolk/cell interface to facilitate the diffusion of injected molecules into the cell portion. The recognition of detailed embryo structures takes 45 ms on the host computer.

Pre-processing is conducted to obtain de-noised binary images. An image is first convolved with a low-pass Gaussian filter for noise suppression. The gray-level image is then binarized to a black-white image using an adaptive thresholding method, in which a local threshold for each pixel is set to be the mean value of its local neighbors. The binary image is eroded to remove small areas that represent spurious features and then, dilated to connect broken segments that originally belong to one object. An example after pre-processing is shown in Figure 6A.

Of the connected objects in the binary image, the one with the maximum area is recognized as the chorion. In Figure 6B, the chorion is enclosed by its minimum enclosing circle. The second largest object in the image is the cytoplasm, the boundary of which is represented by a chain code contour. The boundary of the cytoplasm is often not fully connected (Figure 6A); however, a fully closed contour is important for the recognition of detailed cytoplasm structures including the yolk, the cell portion, and the yolk-cell interface. Thus, a convex hull [22] of the contour is constructed and used as initial positions for subsequent snake tracking [23]. Snakes, or active contours, are often used to locate object boundaries and track deformable objects. They are energy minimizing splines influenced by external constraint forces and image forces that guide snake points towards features such as lines and edges. The closed cytoplasm contour resulting from snake tracking is shown in Figure 6B.

The centroid of the contour, *O* is recognized as the cytoplasm center. The *switching* point, *S* is then determined as the intersect point of the minimum enclosing circle and the horizontal line passing through the cytoplasm center.

In order to distinguish the yolk from the cell portion to provide the flexibility for choosing a desired destination, the cytoplasm contour after snake tracking is fitted into an ellipse using a least squares method, and intercepted into two parts by the minor axis of the fitted ellipse. Based on the fact that the cell portion always has greater convex deficiency [22], the cell and yolk portions are distinguished. The recognized yolk/cell interface is shown in Figure 6B.

## Results

The collected embryos were spread on the surface of the embryo holding device together with embryo media. Applied negative pressure promptly immobilized individual embryos on top of each through-hole. The extra embryos were flushed off the device. The process of embryo immobilization was manually conducted, taking approximately 6–12 s.

The automated system continuously injected a total of 350 zebrafish embryos with fluorescent dyes and 210 embryos with ntl-MO, demonstrating an operation speed of 15 embryos/minute. The injection experiments were arranged in different mornings in a half-a-year period. Each morning, one or two batches of zebrafish embryos were injected. Normally, there were 25 embryos for each batch. The only exception was the first batch for ntl-MO injection with 10 embryos injected for trial.

**Figure 7:**
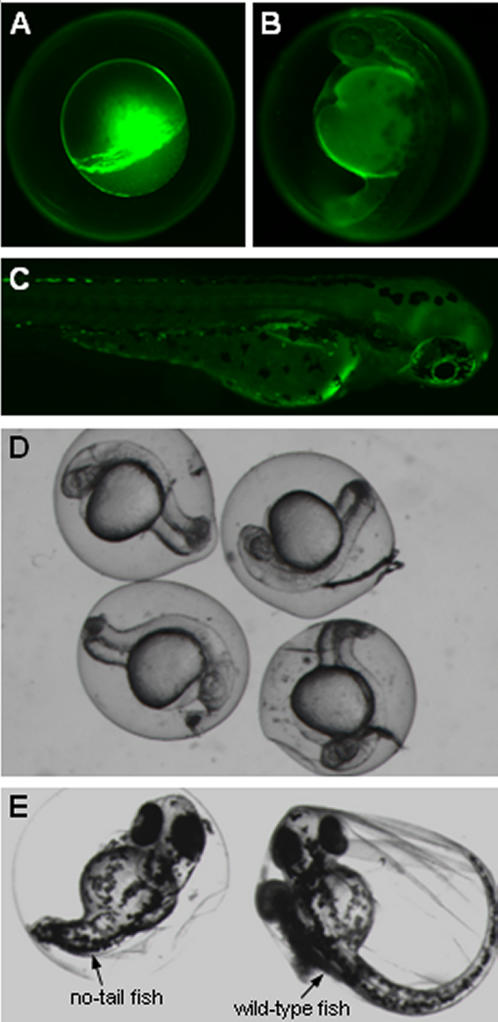
Development of zebrafish embryos injected with fluorescent dyes and ntl-MO. A-C show embryos injected with fluorescent dyes, D-E show embryos injected with ntl-MO. Dye injected embryos are shown immediately following injection (A), 24 hr after injection (B), and 48 hr after injection (C). (D) Ntl-MO injected embryos 24 hr following injection. (E) Comparison of ntl-MO injected embryo (left) with uninjected control embryo (right) 48 hr following injection.

The injected embryos were cultured at 32°C. To determine survival rate and success rate (defined later), embryos injected with fluorescent dyes were inspected under a fluorescence microscope (IX81, Olympus). The embryos were excited by 540 nm laser light and observed through a TRITC filter set. Visual inspection was conducted right after injection, 24 hr after injection, and 48 hr after injection. To quantitate phenotypic rate (defined later), the embryos injected with ntl-MO were inspected under a bright-field microscope (SZX12, Olympus) 24 hr after injection and 48 hr after injection. Figure 7 shows the injected embryos and their subsequent development. The deposited fluorescent dyes (high-brightness) can be clearly observed in the area of the cytoplasm center, as shown in Figures 7A and 7B. Diffused fluorescent dyes are observable 48 hr after injection, as shown in Figure 7C. Bright-field images of four no-tail fishes (24 hr after injection) are shown in Figure 7D. Figure 7E shows a comparison of a no-tail fish 48 hr after injection and control (wild-type).

To quantitatively evaluate the performance of the microrobotic injection system, three measures were defined. (1) *Survival rate:* This measure is defined as the ratio between the number of injected embryos that are capable of developing into larva and the total number of embryos injected, essentially representing the severity and frequency of cell damage from injection. With less cell damage caused in injection, the embryo is more likely to survive and develop normally to a fish. Based on the 350 injected zebrafish embryos, the microrobotic injection system produced an overall survival rate of 98±2%. (2) *Success rate:* This measure is defined as the ratio between the number of embryos with materials successfully deposited in the cytoplasm center and the total number of injected embryos. Essentially, this measure represents the reliability and the reproducibility of the system. It differs from survival rate in that it evaluates the correctness of locating the desired deposition destination. Visual inspection demonstrated that the overall success rate of the 350 injected embryos was 99±1%. (3) *Phenotypic rate:* This measure is defined as the ratio between the number of 48 hour-old embryos demonstrating a no-tail phenotype and the number of embryos with fluorescein-tagged ntl-MO deposited in the cytoplasm center. Essentially, this measure represents the readiness of the system for genetic studies. Based on the 210 ntl-MO injected embryos, the overall phenotypic rate was 98.5±1%. The detailed statistics of the three measures are given in Tables 1 and 2, demonstrating a high degree of reproducibility.

**Table 1:** Statistics of fluorescent dye injection.

Experiment	1	2	3	4	5	6	7	8	**Overall**
# of injected embryos	25	25	50	50	50	50	50	50	**350**
# of survived embryos	24	25	49	48	50	50	48	49	**343**
# of successful injection	25	25	49	49	50	49	49	50	**346**
Survival rate (%)	96	100	98	96	100	100	96	98	**98±2**
Success rate (%)	100	100	98	98	100	98	98	100	**99±1**

**Table 2:** Statistics of ntl-MO injection.

Experiment	1	2	3	4	5	**Overall**
# of injected embryos	10	50	50	50	50	**210**
# of no-tail fishes	10	49	49	49	49	**206**
Phenotypic rate (%)	100	98	98	98	98	**98.5±1**

## Discussion

The operation speed of the automated system (15 embryos with unremoved chorion per minute) compares favorably with the speed of manual injection (10–20 embryos/minute). The embryo holding device permits the completion of immobilizing zebrafish embryos into regular patterns within seconds while manually pushing embryos into agarose trenches, as in the state-of-the-art zebrafish embryo injection, costs minutes.

The achieved survival rate of 98% is consistent with the best survival rate achieved by proficient injection technicians. However, the system is immune from large variations in the survival rate that can reach as low as 70% in manual operation, due to technician fatigue and proficiency differences across technicians. The high survival rate results from efforts in minimizing embryo lysis, by fine-tuning parameters such as the micropipette tip size (∼10 µm), suction pressure (2–7 InHg), injection speed (2.1 mm/s), and retraction speed (4.1 mm/s), which were determined from trials on another 300 zebrafish embryos during system development.

The achieved success rate of 99% demonstrates that the automated system is capable of repeatedly depositing materials at a desired destination inside zebrafish embryos. Rare failures of penetrating an embryo occur when an embryo having drastically different mechanical properties is pushed away during injection. The achieved high reproducibility results from the recognition of embryo structures and precise motion control. The ability of precisely depositing materials at a desired location in a highly reproducible manner has important implications. The elimination of length variations in diffusion paths would make the results of molecule screening more countable.

The 98.5% phenotypic rate of ntl-MO zebrafish embryo injection is consistent with previously reported data using manual injection [18]. The high percentage of mutant phenotypes confirms that Ntl protein is specifically reduced in the microrobotically injected embryos, demonstrating that the automated microrobotic system is a reliable tool for determining new gene functions and more generally, for facilitating large-scale molecule screening.

### Conclusions

Leveraging computer vision and microrobotic control, the high-throughput automated cell injection system experimentally demonstrated the capability of injecting 15 zebrafish embryos per minute with a 98% survival rate, a 99% success rate, and a 98.5% phenotypic rate. The vacuum-based embryo holding device is capable of immobilizing a large number of embryos into regular patterns within seconds, dramatically shortening the sample preparation process. The recognition of embryo structures and precise motion control enable the automated system to precisely deposit a pre-specified amount of materials at a desired destination within the embryo. The application of the microrobotic cell injection system, which is autonomous in operation, fast in speed, free from fatigue, and provides unparalleled reproducibility, to biological and pharmaceutical research for timely injecting materials into a larger number of cells will facilitate large-scale screening of biomolecules or drug compounds. Despite size and property differences among different cell lines, the sample preparation technique and microrobotic control method are applicable to other injection applications such as the injection of mouse oocytes/embryos, *Drosophila* embryos, and other types of suspended cells.
